# Supplementary material for: A Novel Zn2Cys6 Transcription Factor, TopC, Positively Regulates Trichodin A and Asperpyridone A Biosynthesis in Tolypocladium ophioglossoides
Source: Microorganisms. 2023 Oct 17;11(10):2578. doi: 10.3390/microorganisms11102578 (PMC10609478; doi:10.3390/microorganisms11102578)
Supplement: Supplementary file 1 [file microorganisms-11-02578-s001.zip › microorganisms-2567846-supplementary.pdf]

A Novel Zn_2Cys_6 Transcription Factor, TopC, Positively Regulates Trichodin A and Asperpyridone A Biosynthesis in *Tolypocladium ophioglossoides*

Xiang Liu [1,2], Rui-Qi Li [1,2], Qing-Xin Zeng [3], Yong-Quan Li [2] and Xin-Ai Chen [1,2,*]

[1]School of Medicine and the Children's Hospital, Zhejiang University, Hangzhou 310058, China; 22018007@zju.edu.cn (X.L.); 21918020@zju.edu.cn (R.-Q.L.)

[2]Institute of Pharmaceutical Biotechnology, Zhejiang University, Hangzhou 310058, China; lyq@zju.edu.cn

[3]Sir Run Run Shaw Hospital, Zhejiang University School of Medicine, Hangzhou 310058, China; 3415018@zju.edu.cn

*Correspondence: biolab@zju.edu.cn; Tel.: +86-571-8820-8569

Supplementary Tables: **Page**

Table S1. Strains used in this work S4

Table S2. Plasmids used in this study S4

Table S3. Primers used in this study S5

Table S4. Putative annotation of genes in *top* cluster S8

TableS5. HR-ESI-MS data for isolated compounds S9

Table S6. NMR data of F-14329 S10

Table S7. NMR data of trichodin A S11

Table S8. NMR data of asperpyridone A S12

Table S9. NMR data of chaunolidine B S13

Table S10. NMR data of pyridoxatin S14

Supplementary Figures: **Page**

Figure S1. The map of vector using in this study S15

Figure S2. *top* gene cluster and its homogenous clusters S15

Figure S3. Predicted domain of TopC protein S15

Figure S4. The broths of WT and *topC*OE strains after fermentation for 12 days S16

Figure S5. ^{1}H NMR spectrum of **1** (tolypoalbin) in DMSO-d_6 (600 MHz) S17

Figure S6. HRESIMS report of **1** (tolypoalbin) S17

Figure S7. ^{1}H NMR spectrum of **2** (F-14329) in DMSO-d_6 (600 MHz) S18

Figure S8. ^{13}C NMR spectrum of **2** (F-14329) in DMSO-d_6 (150 MHz) S18

Figure S9. HRESIMS report of **2** (F-14329) S19

Figure S10. ^{1}H NMR spectrum of **3** (trichodin A) in DMSO-d_6 (600 MHz) S19

Figure S11. HRESIMS report of **3** (trichodin A) S20

Figure S12. ^{1}H NMR spectrum of **4** (asperpyridone A) in DMSO-d_6 (600 MHz) S20

Figure S13. ^{13}C NMR spectrum of **4** (asperpyridone A) in DMSO-d_6 (150 MHz) S21

Figure S14. HRESIMS report of **4** (asperpyridone A) S21

Figure S15. ^{1}H NMR spectrum of **5** (chaunolidine B) in DMSO-d_6 (600 MHz) S22

Figure S16. ^{13}C NMR spectrum of **5** (chaunolidine B) in DMSO-d_6 (150 MHz) S22

Figure S17. HRESIMS report of **5** (chaunolidine B) S23

Figure S18. ^{1}H NMR spectrum of **6** (pyridoxatin) in DMSO-d_6 (600 MHz) S23

Figure S19. ^{13}C NMR spectrum of **6** (pyridoxatin) in DMSO-d_6 (150 MHz) S24

Figure S20. HRESIMS report of **6** (pyridoxatin) S24

Figure S21. Determination of the *top* cluster borders by RT-PCR S25

Figure S22. PCR amplification with different probes. M: Generuler Marker. S25

Figure S23. Deletion of *topH* in *topC*OE S25

Figure S24. HRESIMS report of **7** (tolypyridone C) S26

Figure S25. HRESIMS report of **12** S26

Figure S26. Deletion of *topA* in *topC*OE. S27

Table S1. Strains used in this study

Strains	Description	source
T. ophioglossoides	WT	This lab
topCOE	Overexpression of *topC* in the *T.ophioglossoides*	This study
dtopA-Bar	knock out of *topA* in *topC*OE strain	This study
dtopB-Bar	knock out of *topB* in *topC*OE strain	This study

Table S2. Plasmids used in this work

plasmids	Description	source
pFGL815N	E. coli-A. tumefaciens shuttle expression vector,<br>kanamycin marker	This lab
pTefTAS-2P+2T	*T.ophioglossoides* shuttle expression vector,<br>containing *sur* marker	This study
pTefTAS*topC*	Overexpression of *topC* in *T.ophioglossoides* WT	This study
d*topB*-Bar	knock out of *topB* in *topC*OE	This study
d*topA*-Bar	knock out of *topA* in *topC*OE	This study

Table S3. Primers used in this study

Name	Oligonucleotide sequence (5'-3')	Uses
g9685-R(SpeI)	CTACTACAGATACTAGTTCAGTCAAGCCATCCTTCGCC	For *topC* overexpression
g9685-F(SpeI)	ACAACCGTCAAACTAGTATGAGTACACAATCAAGCCAGGGC	
pApTEF1-F(HindIII)	TGCAGGTCGACTCTAGAAGCAACAGGCCAGGCTAGACG	
pApTEF1-R(fusion)	AACTGGTCATTTCTAGATTGACGGTTGTGTATGGAAGATTGAGTG	
pApTEF-2-R(fusion)	ACTAGTTTGACGGTTGTGTATGGAAGATTGAGTG	
T-amyB2-F(fusion)	ACAACCGTCAAACTAGTATCTGTAGTAGCTCGTGAAGGGTG	
sur-F(fusion)	attataggaaaggtaccTCGACGTGCCAACGCCACAG	
sur-R(EcoRI)	CTCGAGaattccgacgtgagagcatgcaattcc	
qrt-TEF-F	ATCAAGGAGAAGATCGACCGCC	qRT-PCR primers for *top* cluster
qrt-TEF-R	ACGGACTTGATGACACCGACG	
qrt-g9690-F	CGCGTTAACTACAACGTGCCG	
qrt-g9690-R	TAGCGAAGTATGGCGCTTCGG	
qrt-g9689-F	GTTCTCGAGGTCGACCTGCG	
qrt-g9689-R	TCACGACCGAGGCCAGTTATGTC	
qrt-	TCTGTGCCGGGATCTTGTAGCG	

g9688-F		
qrt-g9688-R	ACCAAGCATCCCGACTTCCAAC	
qrt-g9687-F	AGTGGATCCATGCCTCCCTTG	
qrt-g9687-F	AGTGGATCCATGCCTCCCTTG	
qrt-g9687-R	GGAAGCACCATGGCAACATTAGC	
qrt-g9686-F	GGGTCCAGACCCATGTACTTGC	
qrt-g9686-R	ATGATTCGCAAGTCGGGACTCG	
qrt-g9685-F	AGCATGTCCATCGACACTGCTAG	
qrt-g9685-R	TGCTCGTACTTTGTCCCTCGG	
qrt-g9684-F	CCTGCACGATGAAGGTTAGGTCC	
qrt-g9684-R	GTGGAACGGCATCAAGGGCAAG	
qrt-g9683-F	GTGCCGTCTTGGATGTTCCG	
qrt-g9683-R	GCCCGACTTCGATCCTGTCTTC	
pFG-pApTEF-F(SpeI)	cccggggatccactagtagcaacaggccaggctagac	for construction of deletion strains
pFG-TamyB-R(PstI)	AGCTTgcatgcctgcagtttcctataatagactagcgtgcttg	
pApTEF-R(fusion)	TTCTGGGCTCATtgacggttgtgtatggaagattgagtg	
TamyB-F(fusion)	GTCACCGAGATCTGAatctgtagtagctcgtgaagggtg	
Bar-F(fusion)	cttccatacacaaccgtcaATGAGCCCAGAACGACGCCC	
Bar-R(fusion)	acgagctactacagatTCAGATCTCGGTGACGGGCAG	
5’d*topA*-F(SpeI)	cccggggatccactagtaagatgttgtagcgggccagg	
5’dtopA-R(SpeI)	ggcctgttgctactagtttcggaagacttttagtgctgaaccg	
3’d*topA*-	attataggaaactgcagtctagtgtccttctccaagggcg	

F(PstI)		
3’d*topA*-R(PstI)	AGCTTgcatgcctgcagccgcttccgttctatcgaggtac	
5’d*topB*-F(SpeI)	cccggggatccactagtgtgcttgttgcccgacttgg	
5’d*topB*-R(SpeI)	ggcctgttgctactagtcttcagtatctgcacacgtgcg	
3’d*topB*-F(PstI)	attataggaaactgcagtgagcgagcactgtgcccgc	
3’d*topB*-R(PstI)	AGCTTgcatgcctgcagggttatacgggccgagtcactg	
g9685-DBD-F(EcoRI)	ATATCGGATCCGAATTCATGAGTACACAATCAAGCCAGGGC	EMSA experiment
g9685-DBD-R1(EcoRI)	CGACGGAGCTCGAATTCGGCATTATCGTAGCTGGCTGATG	
AB-Promter-F-FAM	ccccataatgggaacgcgttg	
AB-Prpmter-R	aacgaaggtcttgagatgcaacaatcg	
CD-Promter-F-FAM	acattgtatctcgggattggaacg	
CD-Promter-R	tgtctccatttgatgacagactaccc	
E-Promter-F1	ggtgtatgagctttgagacgggctc	
E-promoter-R-FAM	gtctctcgcctaggcatcacgc	
FG-Promter-F-FAM	cttcagtatctgcacacgtgcg	
FG-Promter-R	cggaagacttttagtgctgaaccgtg	
dg9684-F	gatggaggaactgaatgcccg	PCR verification
dbar-R	GGTTGACGATGGTGCAGACCG	

dbar-F1	GGCAGCTGGACTTCAGCCTG	of deletion strains
dg9684-R1	agccttggccggtcacttgc	
dg9683-F	gtcgctgataacgctgttctcg	
dg9683-R1	gccggcatggtggcttccac	
dg9684-F3	tgacgacgagtcaagtcgacg	
dg9683-R2	agtactctagccctcgcaaagc	
g8899-P-F	ggtatctcgtggtccatagttgcc	
g8899-P-R	gccatgatatgcggcaagtgttg	

Table S4. Putative annotation of genes in *top* cluster

Deduced functions of genes in the *top* biosynthesis gene cluster and protein homologs annotation

Gene	aa	Proposed function	Homologous gene in *tol*, *lep*, *pdx*	Identity/similarity (%)	fpkm
topA	499	cytochrome p450	pdxF	80.8/89.6	0.091456417
topB	459	O-methyltransferase	lepI, pdxI (DA); lepF, pdxG (SDR)	24.3/37, 85.2/92.6; 20.4/32.9, 65.9/75.1	0.0541275
topC	786	C6 transcription factor	lepE, pdxE	28.7/45.1,20.1/28.3	0.188618
topD	422	enoyl_reductase	tolC, lepG, pdxD	74.8/81, 41.1/59.2, 83.9/87.9	0.0826054
topE	4042	PKS-NRPS	tolA, lepA, pdxC	82.8/90.1, 45.6/62.3, 71.9/81.6	0.0225088
topF	472	cytochrome p450	tolD, lepD, pdxB	75.6/82.3, 38.8/59, 21.2/38.3	0.13772577

topG	574	cytochrome p450	tolB, lepH	94.4/96.8, 49.8/68.5	0.0608727
topH	225	SDR	pdxG	82/91.7	0.0174218

Table S5. HR-ESI-MS data for isolated compounds

compound	m/z	HRESI-MS	molecular formula	UV
**1**	358.2000	$[M+H]^+$	$C_{21}H_{27}NO_4$	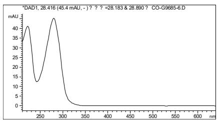
**2**	372.1818	$[M-H]^-$	$C_{21}H_{27}NO_5$	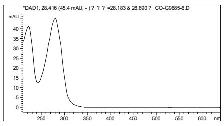
**3**	388.1769	$[M-H]^-$	$C_{21}H_{27}NO_6$	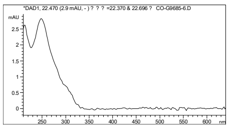
**4**	278.1740	$[M+H]^+$	$C_{16}H_{23}NO_3$	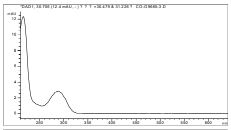
**5**	388.1768	$[M+H]^+$	$C_{21}H_{27}NO_6$	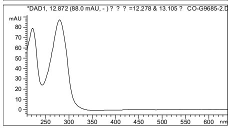
**6a**+**6b**	262.1456	$[M-H]^-$	$C_{15}H_{21}NO_3$	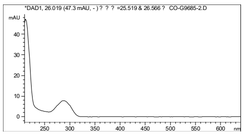
**7**	356.1861	$[M+H]^+$	$C_{21}H_{25}NO_4$	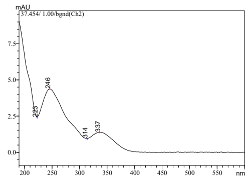
**12**	278.1408	$[M-H]^-$	$C_{15}H_{21}NO_4$	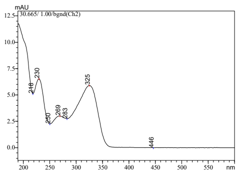

Table S6. ^{1}H (600 MHz, DMSO-d_6) and ^{13}C (150 MHz, DMSO-d_6) NMR data of F-14329

Position	δ_H (mult, J (Hz))	δ_C
1		176.0
2		100.6
3		191.5
4	3.49 (br m)	33.4
5a	1.62 (m)[b]	39.8[d]
5b	1.04 (m)	
6	1.24 (m)	30.8
7a	1.86 (m)	39.9[d]
7b	1.74 (m)	
8	5.33 (m)[a]	129.3[c]
9	5.34 (m)[a]	125.9
10	1.59 (d, 5.2)[b]	
11	0.88 (d, 6.3)	18.1
12	0.77 (d, 6.3)	19.1
1’		192.6
2’	4.16 (br s)	68.0
3’	4.87 (br s)	72.6
4’		129.3[c]
5’/9’	7.00 (d, 8.5)	128.2
6’/8’	6.59 (d, 8.5)	114.1
7’		156.5
3’-OH	5.65 (br s)	
7’-OH	9.23 (br s)	
-NH	9.14 (br s)	

[a, b, c] Overlapping signals; [d] Overlapped with residual DMSO signal

Table S7. ^{1}H (600 MHz, DMSO-d_6) NMR data of trichodin A

Position	δ_H (mult, J (Hz))
1	-
2	-
3	2.62 (dd, 10.5, 10.3)
4	1.72 (m)
5α	1.05 (m)
5β	1.70 (m)
6	1.60 (m)
7α	1.16 (m)^b
7β	1.40 (m)^a
8	1.37 (m)^a
9	3.67 (m)
10	1.20 (d, 6.2)^b
11	1.06 (d, 6.9)
12	0.93 (d, 6.7)
1’	-
2’	6.99 (s)
3’	-
4’	-
5’/9’	7.17 (d, 8.5)
6’/8’	6.72 (d, 8.5)
7’	-
7’-OH	10.93 (br s)
-NH	9.37 (br s)

$^{a, b}$ Overlapping signals;

Table S8. ^{1}H (600 MHz, DMSO-d_6) and ^{13}C (150 MHz, DMSO-d_6) NMR data of asperpyridone A

Position	δ_H (mult, J (Hz))	δ_C
2		159.3
3		112.5
4		162.5
5	5.80, d (7.7)	98.6
6	7.67, d (7.7)	133.7
7	2.11	43.8
8	1.36	48.4
9a	0.78	36.6
9b	1.75	36.6
10	1.59	32.2
11a	0.95	45.6
11b	1.67	45.6
12	1.55	39.8
13	3.65	77.2
14	1.24	18.7
15	0.91	21.8
16	1.00	22.3
1-OMe	3.84 (s)	64.0

Table S9. ^{1}H (600 MHz, DMSO-d_6) and ^{13}C (150 MHz, DMSO-d_6) NMR data of chaunolidine B

Position	δ_H (mult, J (Hz))	δ_C
1		176.0
2		100.5
3		191.5
4	3.48 (br s)	33.3
5a	1.62 (m)	39.4[b]
5b	1.06 (m)	
6	1.28 (m)	30.7
7a	1.92 (m)	39.7[b]
7b	1.77 (m)	
8	5.48(m)[a]	132.1
9	5.47 (m)[a]	127.9
10	3.85 (d, 5.2)[b]	61.4
11	0.88 (d, 6.3)	18.1
12	0.78 (d, 6.3)	19.1
1’		192.6
2’	4.16 (br s)	68.0
3’	4.87 (br s)	72.6
4’		129.3[c]
5’/9’	7.00 (d, 8.5)	128.2
6’/8’	6.59 (d, 8.5)	114.1
7’		156.5
3’-OH	5.64 (br s)	
7’-OH	9.23 (br s)	
-NH	9.15 (br s)	

[a] Overlapping signals; [b] Overlapped with residual DMSO signal

Table S10. ^{1}H (600 MHz, DMSO-d_6) and ^{13}C (150 MHz, DMSO-d_6) NMR data of pyridoxatin

position	Rotamer A (major)[f]		Rotamer B (minor)[f]	
	δ_H	δ_C	δ_H	δ_C
1		157.8		160.3[e]
2		112.1		112.6
3	2.35 (m)[d]	45.5	2.61 (d, 11.2, 11.2)	45.9
4	3.01 (d, 9.0)	41.5	2.78 (d, 11.0)	42.8
5a	1.67 (m)[a]	41.7	1.68 (m)[a]	41.9
5b	0.84 (m)[b]		0.84 (m)[b]	
6	1.54 (m)	31.4	1.54 (m)	31.5
7a	1.67 (m)[a]	44.2	1.67 (m)[a]	44.3
7b	0.67 (m)[c]		0.68 (m)[c]	
8	2.33 (m)[d]	31.3	2.14 (m)	32.5
9	5.48 (m)	143.5	5.48 (m)	143.5
10a	4.73 (dd, 16.4, 1.6)	112.4	4.76 (d, 16.2, 1.6)	112.5
10b	4.64 (dd, 10.2, 1.6)		4.63 (dd, 10.2, 1.6)	
11	0.62 (d, 5.6)[c]	20.5	0.64 (d, 6.6)[c]	20.5
12	0.89 (d, 6.4)[b]	22.7	0.89 (d, 6.4)[b]	22.6
1’		161.2		160.3[e]
2’	7.55 (d, 7.6	131.9	7.50 (d, 7.6)	132.0
3’	5.83 (d, 7.6)	96.3	5.80 (d, 7.6)	97.1
1’-OH	9.97 (br s)		9.97 (br s)	
N-OH	11.06 (br s)		11.06 (br s)	

[a, b, c, d, e] Overlapping signals; [f] The ratio of rotamers A and B of pyridoxatin is approximately 5:3 in DMSO

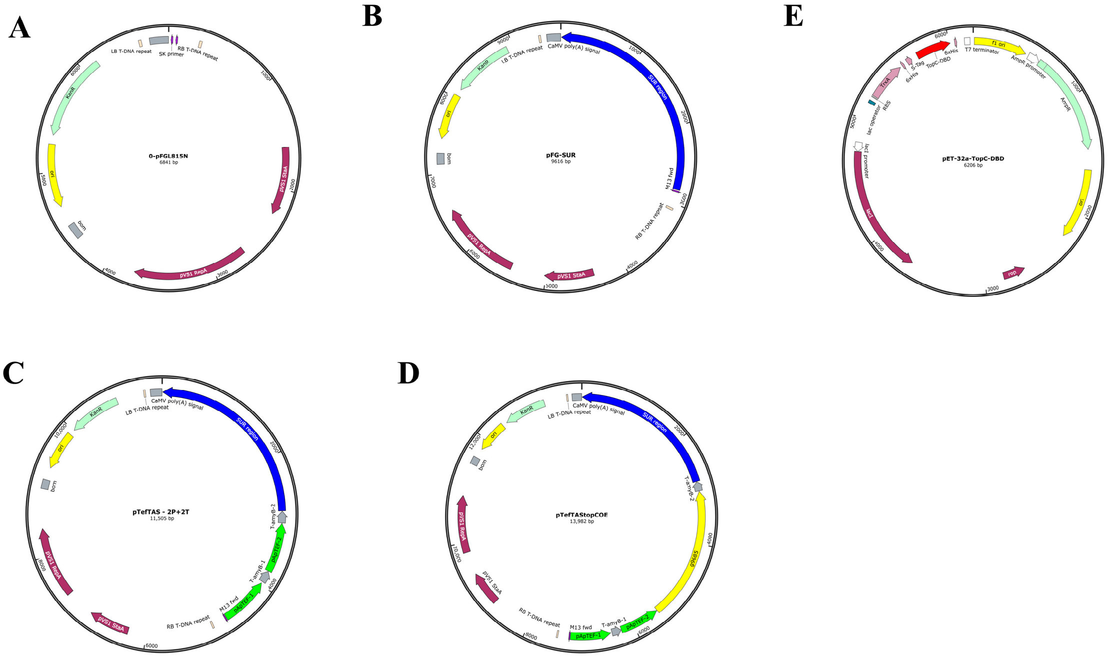

Figure S1. The map of vector using in this study. A. The map of the shuttle plasmid pFG-815N. B. The map of the pFG-SUR. C. The map of the pTEFTAS-2P+2T. D. The map of the pTEFTAS-topCOE. E. The map of the Pet-32a-TopC-DBD

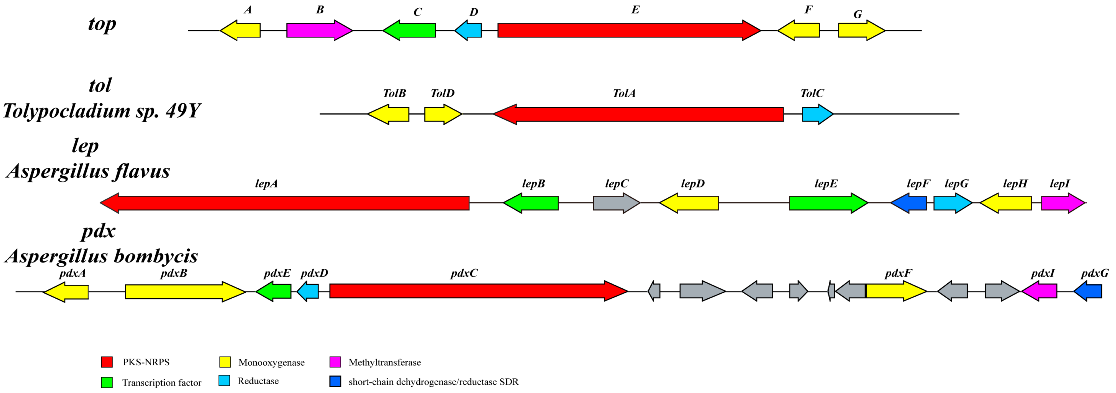

Figure S2. *top* gene cluster and its homogenous clusters

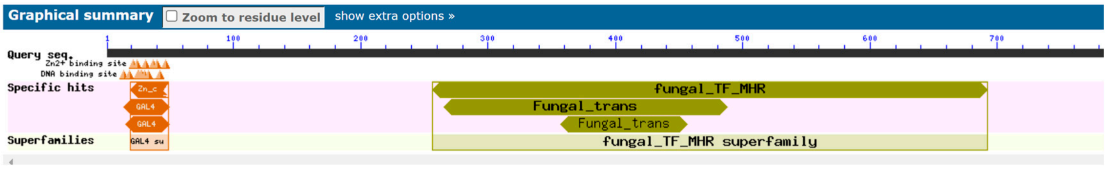

Figure S3. Predicted domain of TopC protein

Chemical analysis

Small scale: the *topC*OE transformants were grown in 100 mL COB medium at 26 ℃ for 12 days with shaking 220 rpm. Then 1 mL culture media was extracted with equal volume of ethyl acetate. And the extract was evaporated to remove ethyl acetate and resolved in 200 μL to detect the metabolites by HPLC.

Isolation of compounds

*topC*OE was cultivated in COB liquid medium (8 L; 800 mL×5) at 26 ℃, shaking at 180 rpm for 14 d. The culture media and mycelium were separated by filtration though miracloth. The culture media was extracted with equal volume of ethyl acetate (repeated three times). The organic layers were combined and concentrated by rotary evaporation. The mycelium was extracted with methanol overnight under the ultrasonic wave state. The extracts were filtrated and concentrated in vacuo. HPLC purification was performed with a semi-preparative reverse-phase C18 column.

HPLC purification conditions: Flow rate: 2.5 mL/min, Solvent gradient system: acetonitrile and water with 0.1% formic acid (0-5 min: 5:95, 6-10 min: 5:95 to 40:60, 11-55 min: 40:60 to 90:10, 56-60 min: 90:10 to 100:0). Absorbance was monitored at 280 nm and 210 nm.

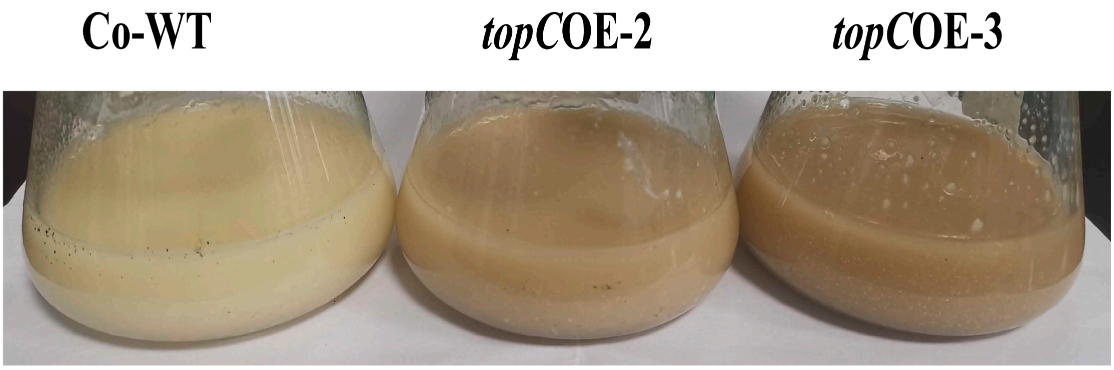

Figure S4. The broths of WT and *topC*OE strains after fermentation for 12 days.

The 1D NMR spectra and HRESIMS of **1-6**

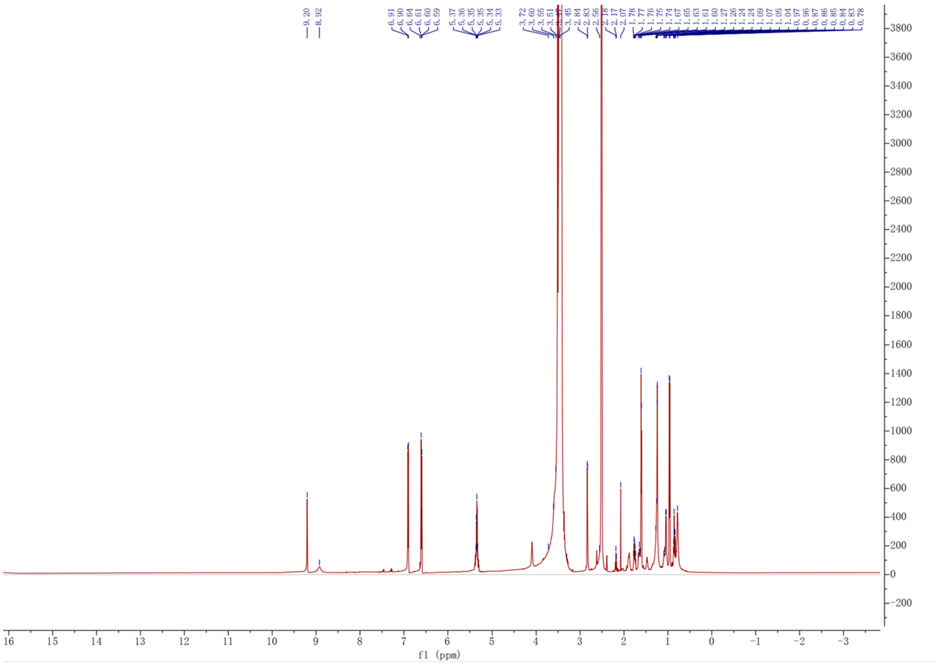

Figure S5. ^{1}H NMR spectrum of **1** (tolypoalbin) in DMSO-d_6 (600 MHz)

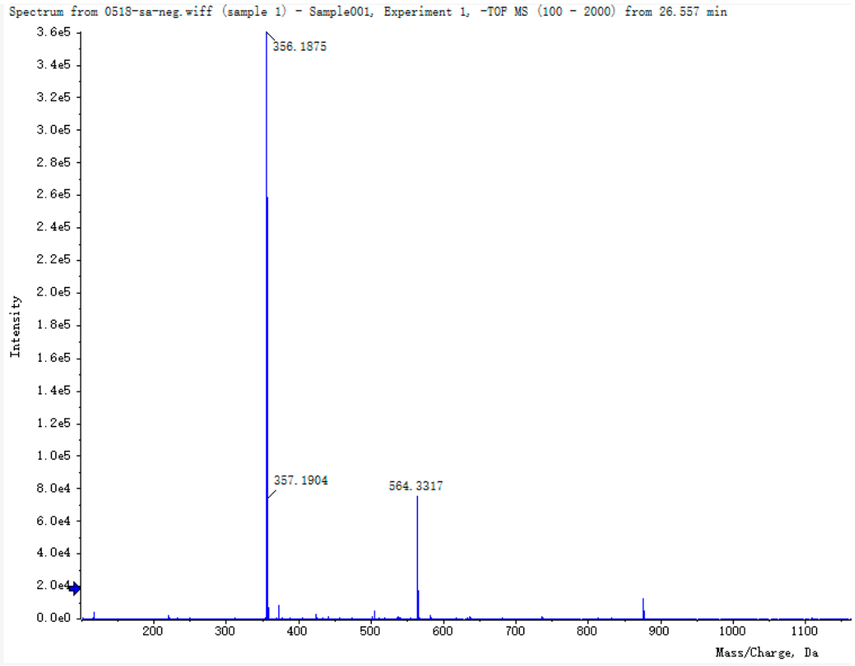

Figure S6. HRESIMS report of **1** (tolypoalbin)

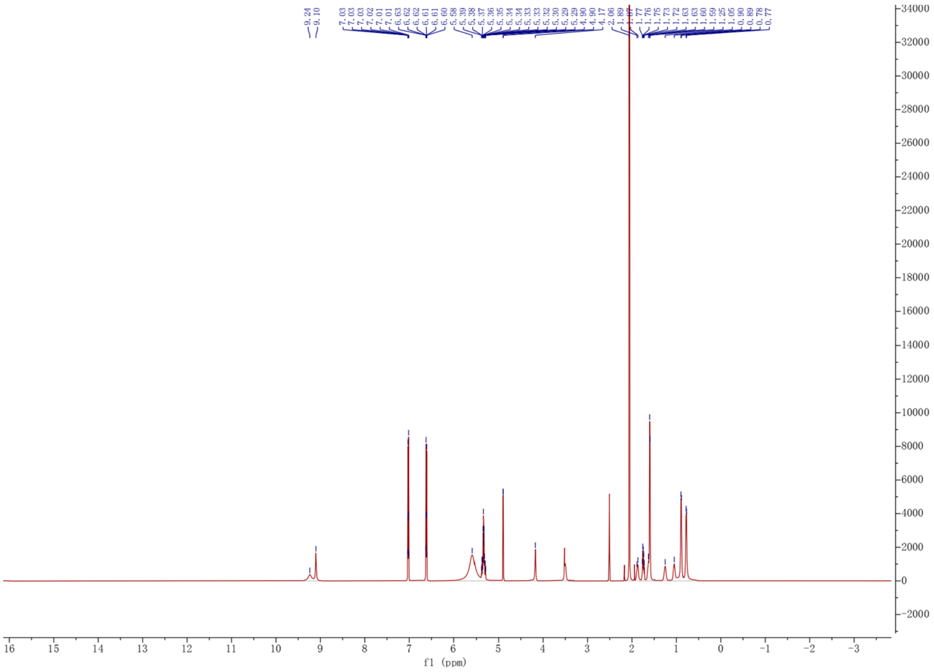

Figure S7. ^{1}H NMR spectrum of **2** (F-14329) in DMSO-d$_6$ (600 MHz)

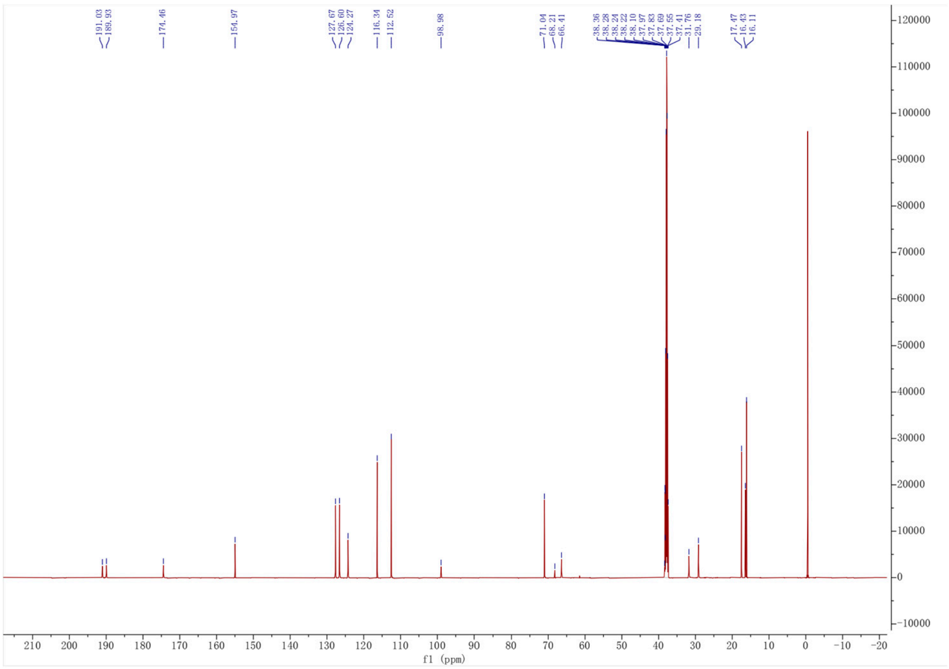

Figure S8. ^{13}C NMR spectrum of **2** (F-14329) in DMSO-d_6 (150 MHz)

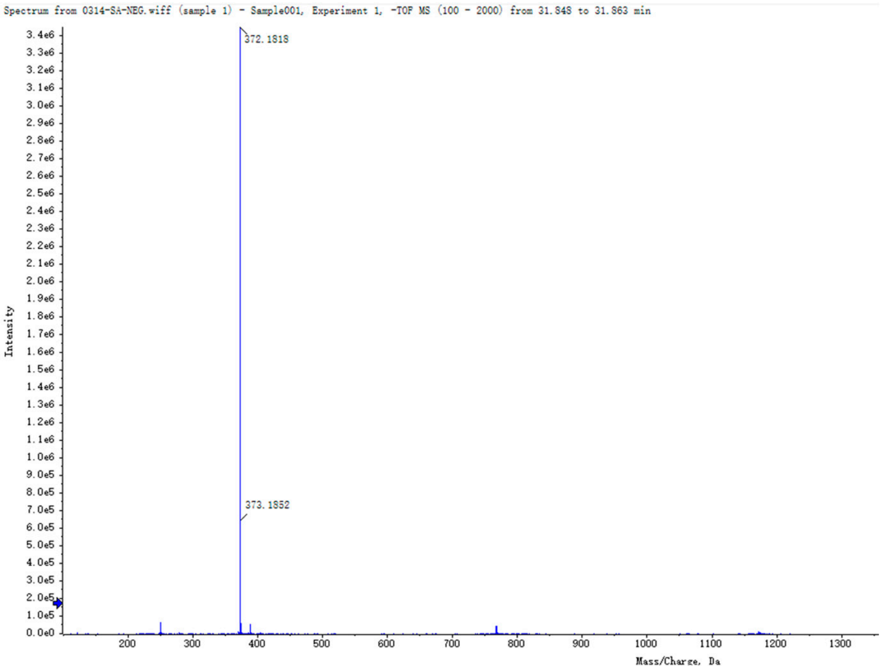

Figure S9. HRESIMS report of **2** (F-14329)

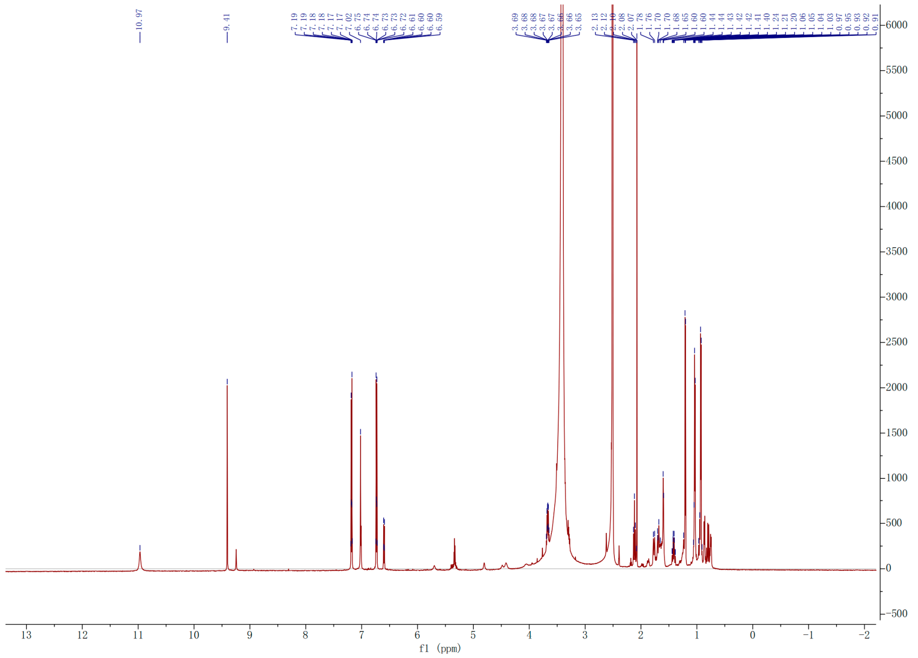

Figure S10. ^{1}H NMR spectrum of **3** (trichodin A) in DMSO-d_6 (600 MHz)

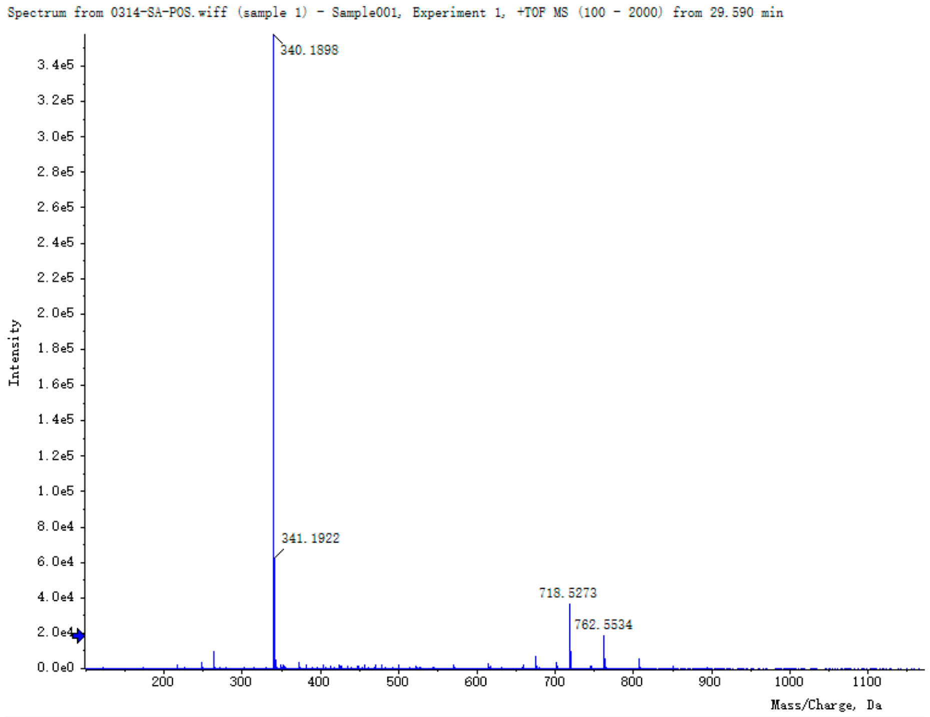

Figure S11. HRESIMS report of **3** (trichodin A)

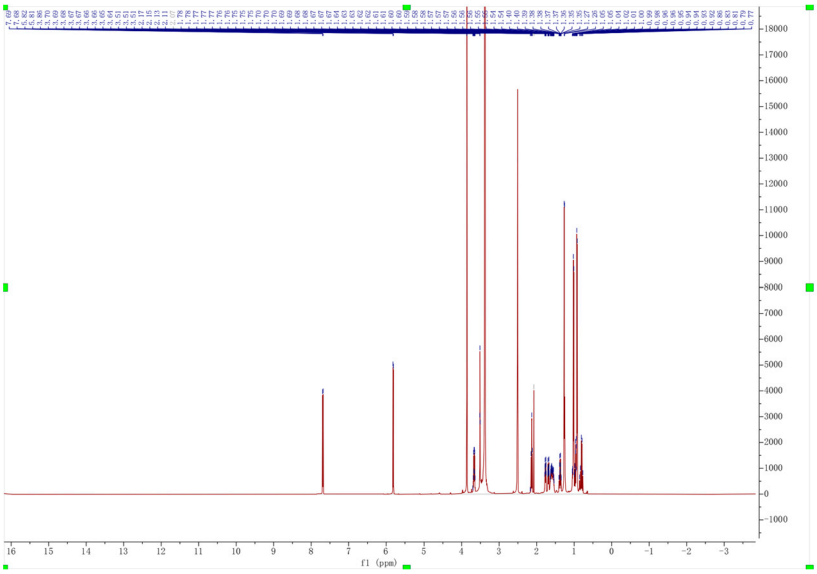

Figure S12. ^{1}H NMR spectrum of **4** (asperpyridone A) in DMSO-d$_6$ (600 MHz)

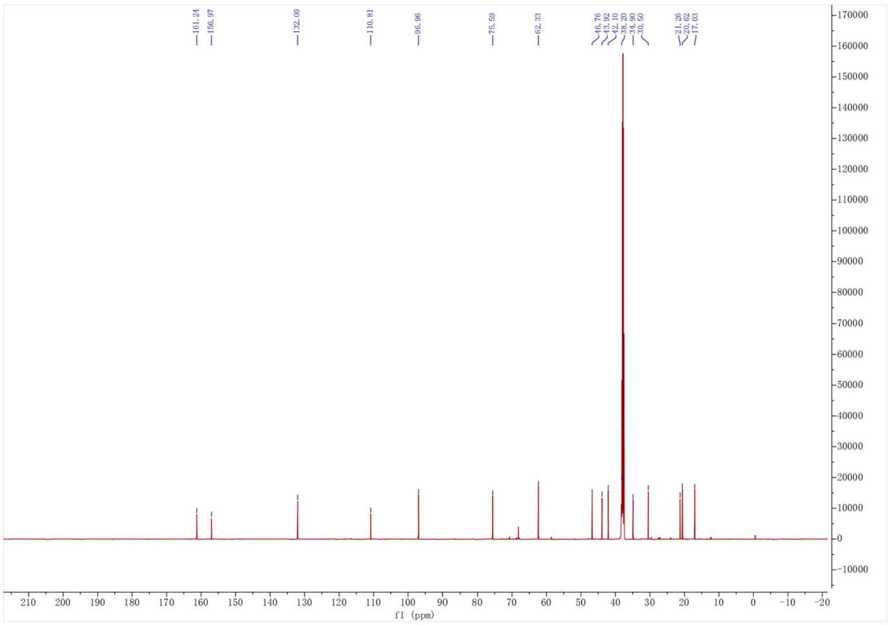

Figure S13. ^{13}C NMR spectrum of **4** (asperpyridone A) in DMSO-d$_6$ (150 MHz)

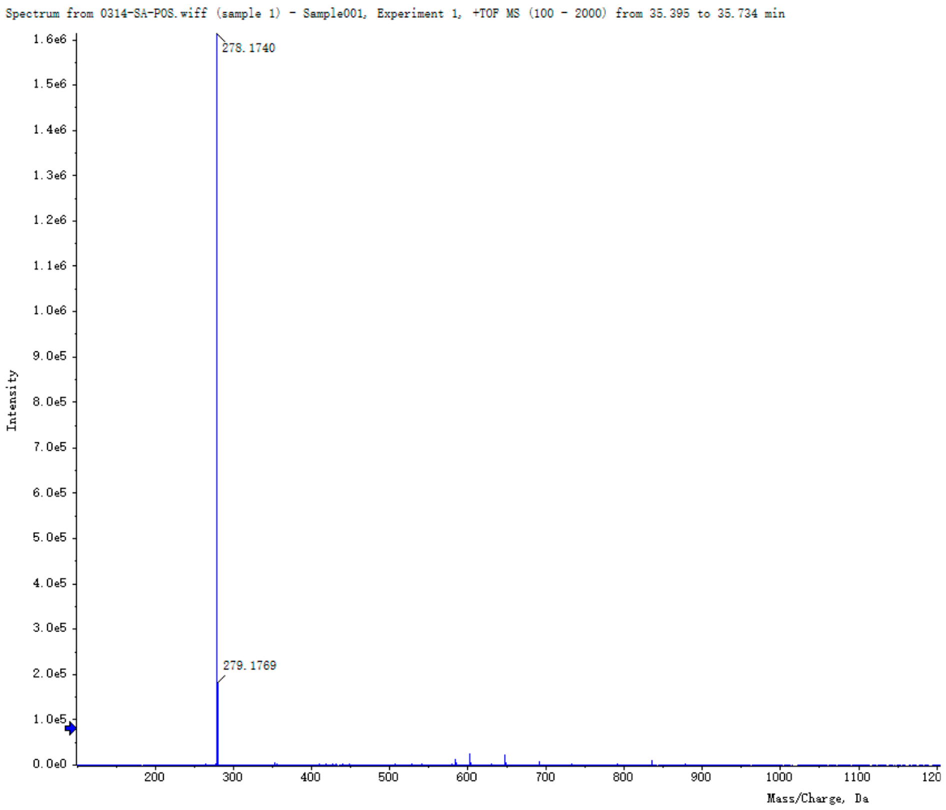

Figure S14. HRESIMS report of **4** (asperpyridone A)

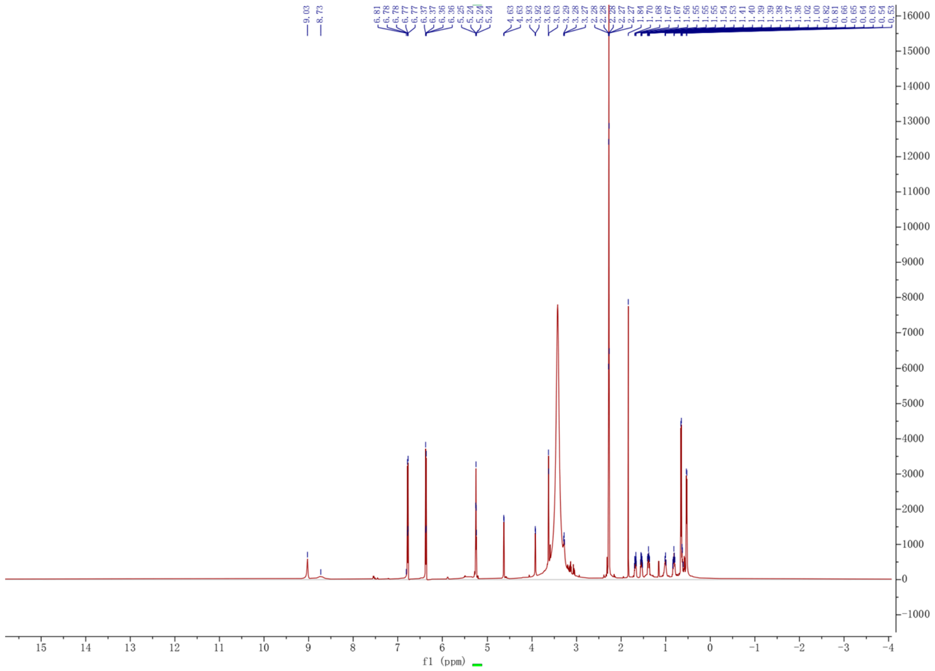

Figure S15. ^{1}H NMR spectrum of **5** (chaunolidine B) in DMSO-d_6 (600 MHz)

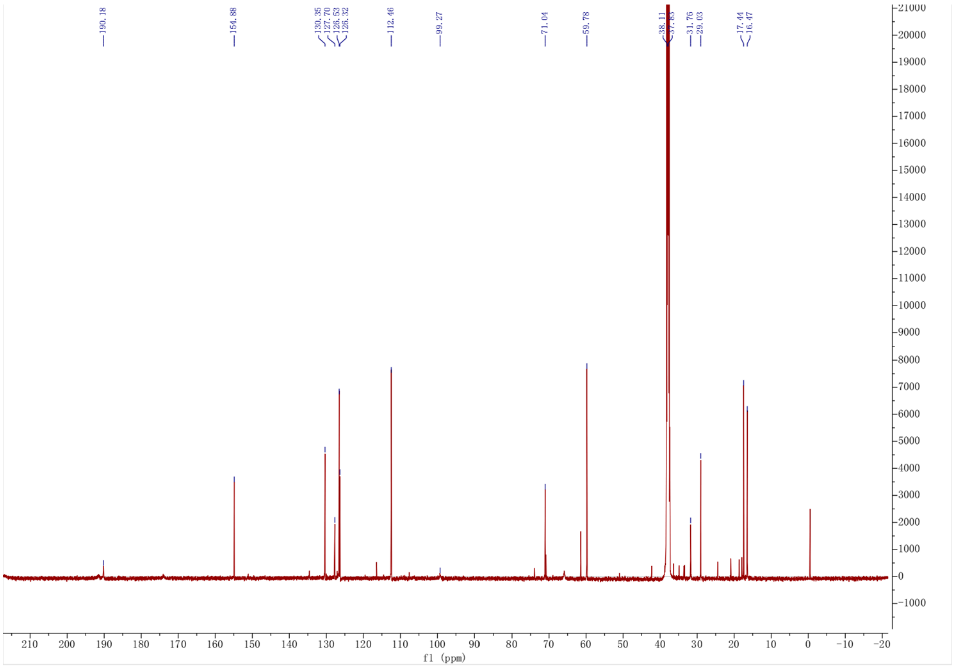

Figure S16. ^{13}C NMR spectrum of **5** (chaunolidine B) in DMSO-d_6 (150 MHz)

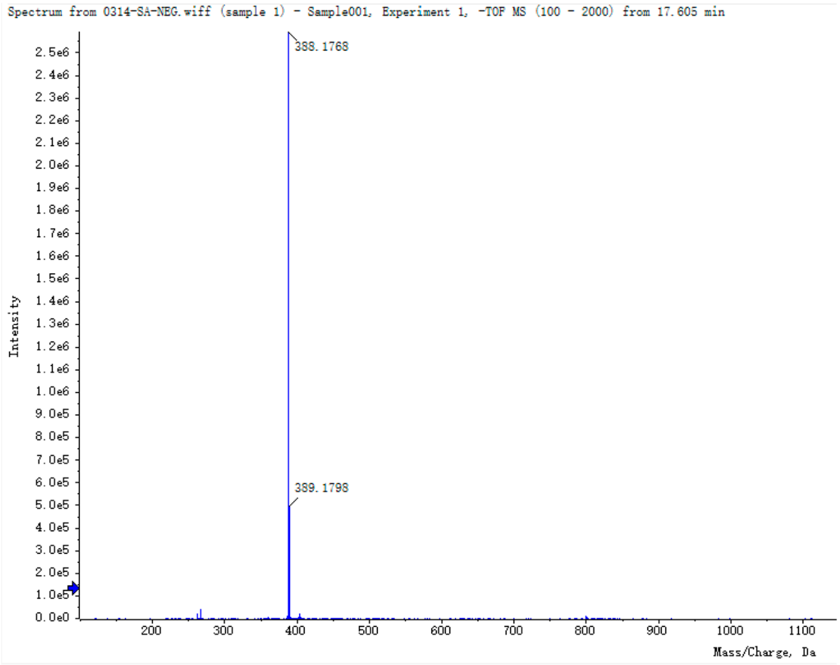

Figure S17. HRESIMS report of **5** (chaunolidine B)

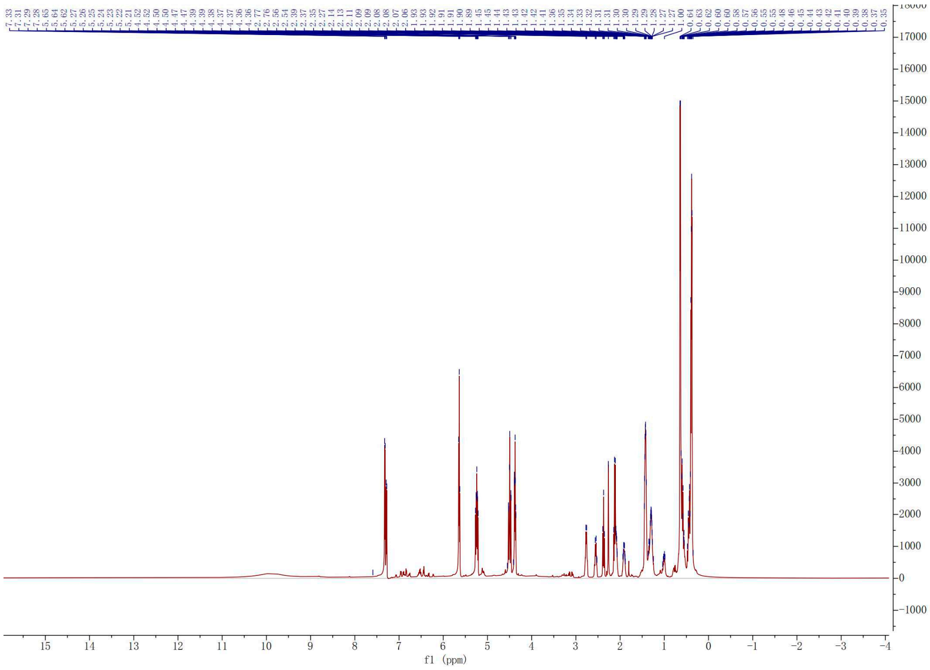

Figure S18. ^{1}H NMR spectrum of **6** (pyridoxatin) in DMSO-d_6 (600 MHz)

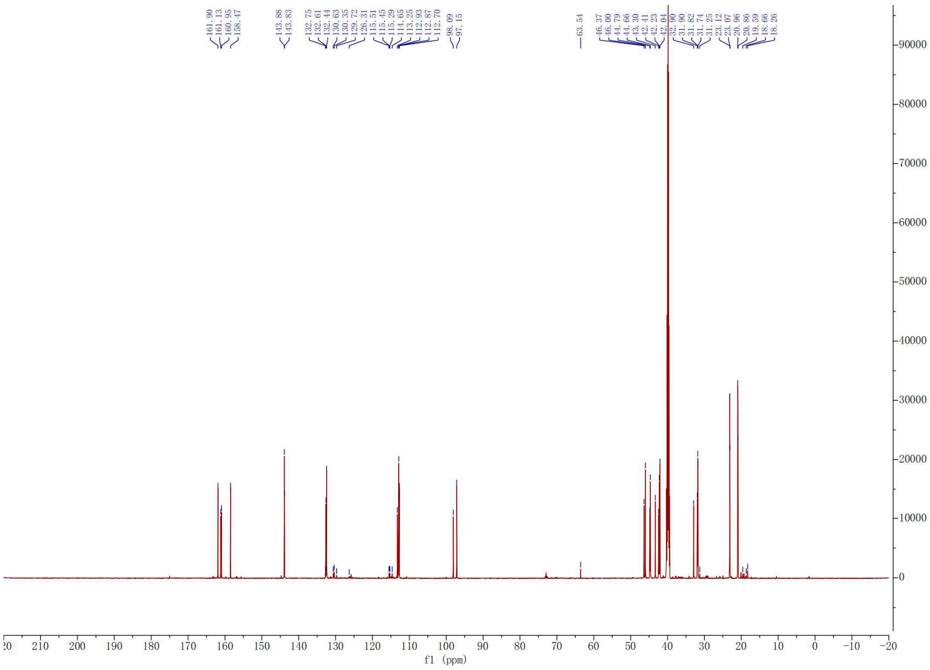

Figure S19. ^{13}C NMR spectrum of **6** (pyridoxatin) in DMSO-d_6 (150 MHz)

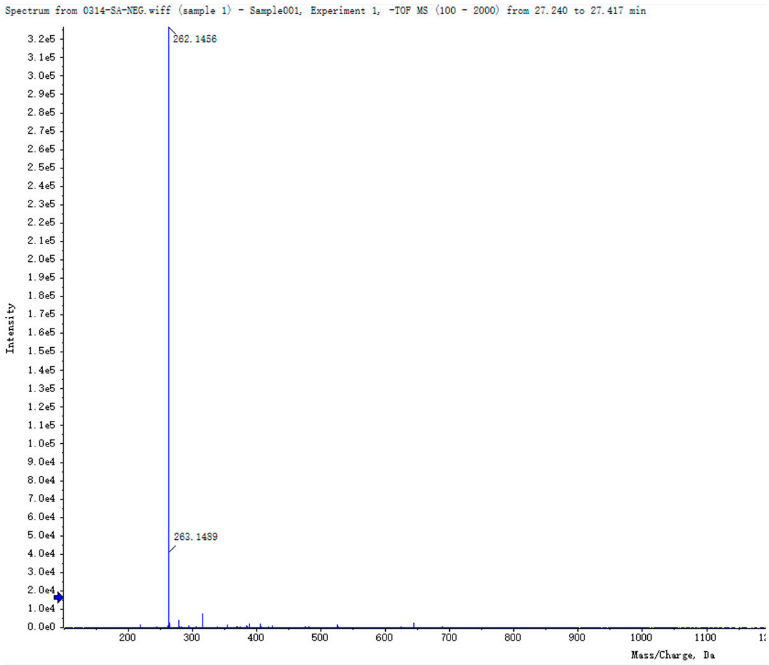

Figure S20. HRESIMS report of **6** (pyridoxatin)

The result of RT-qPCR analysis was shown in the following figure.

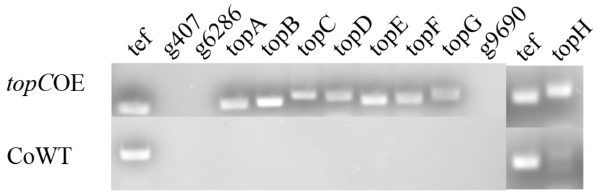

Figure S21. Determination of the *top* cluster borders by RT-qPCR

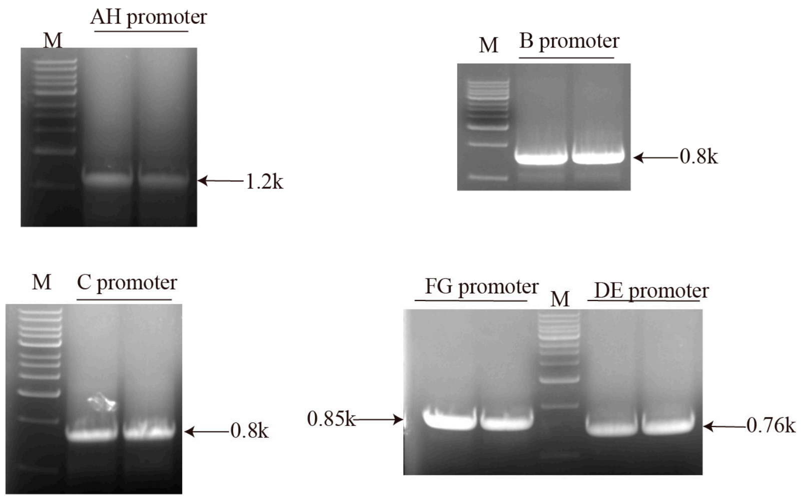

Figure S22. PCR amplification with different probes. M: Generuler Marker.

Deletion of genes in *top*COE strain

The deletion of the target gene based on homologous recombination was performed in *T.ophioglossoides*[1].

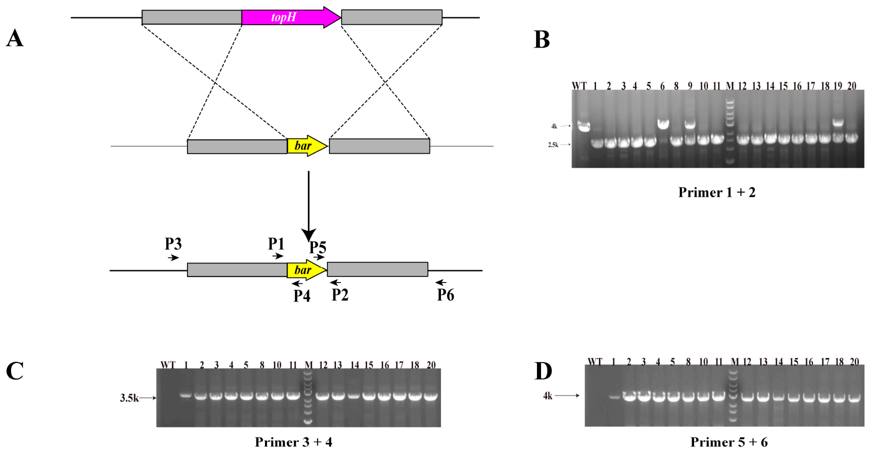

Figure S23. Deletion of *topH* in *topC*OE. The transformants was confirmed by PCR with primers as indicated. Primers1-6 refers to dg9684-F/dg9684-R1/dg9684-F3/dbar-R/dbar-F1/qrt-g9685-F, as shown in Table S3

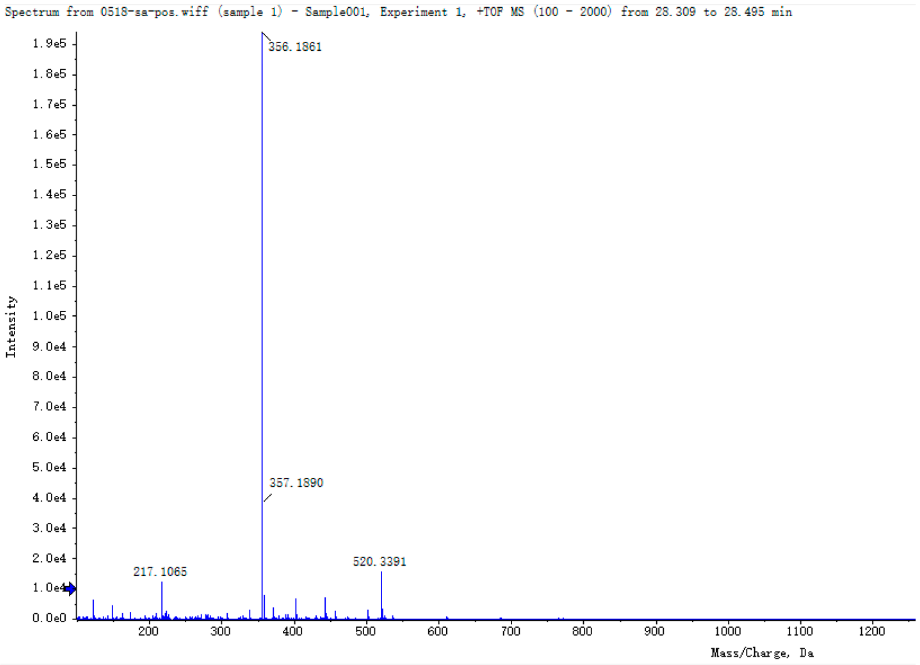

Figure S24. HRESIMS report of **7** (tolypyridone C)

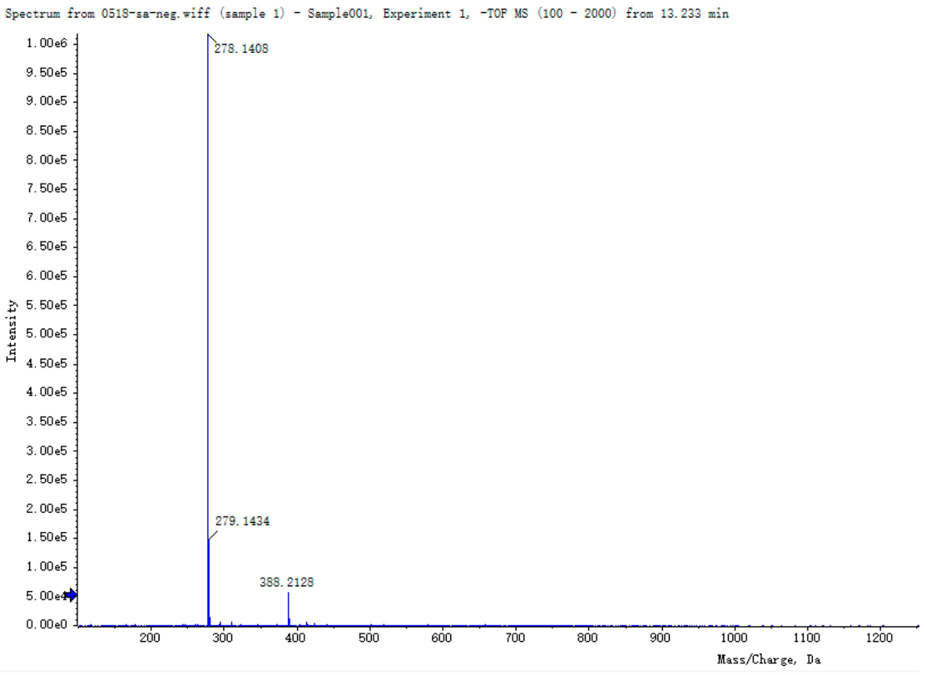

Figure S25. HRESIMS report of **12**

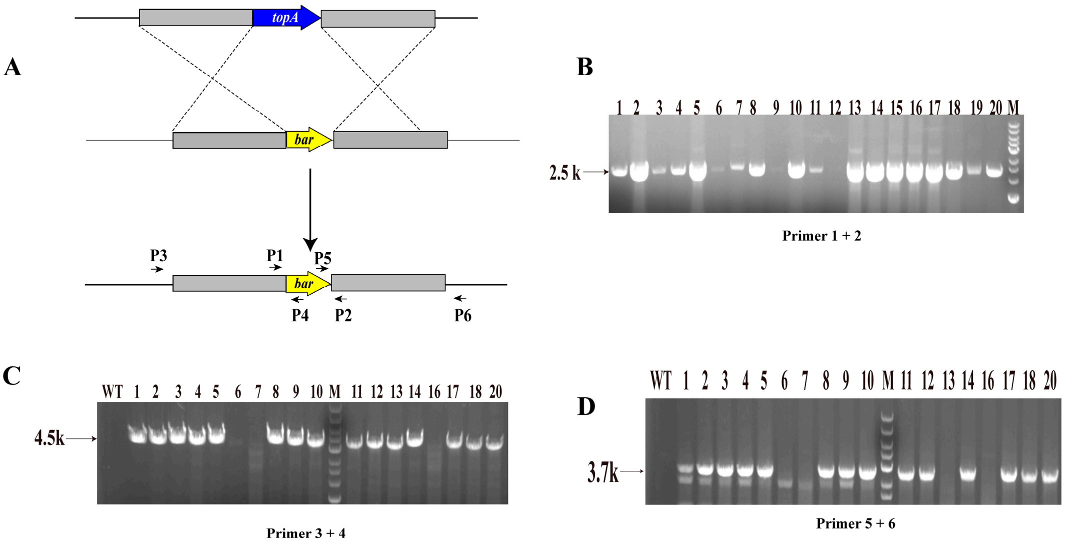

Figure S26. Deletion of *topA* in *top*COE. The transformants was confirmed by PCR with primers as indicated. Primers1-6 refers to dg9683-F/dg9683-R1/qrt-g9684-F/dbar-R/dbar-F1/dg9683-R2, as shown in Table S3

Reference

[1] He, X., M. Zhang, Y. Y. Guo, et al. Revelation of the Balanol Biosynthetic Pathway in *Tolypocladium ophioglossoides* [J].*Org Lett* 2018, **20**(20):6323-6326.
